# Major Depressive Disorder Signatures: A Review of Artificial Intelligence (AI)‐Powered Insights Into Gut Dysbiosis

**DOI:** 10.1002/hsr2.73159

**Published:** 2026-09-07

**Authors:** Nazanin Zahra Keshvari, Sara Asl Motaleb Nejad Sarkhab, Tara Shahmoradi, Mohammad Pourashory, Arash Esmaeili, Kiarash Saleki, Nima Rezaei

**Affiliations:** ^1^ Network of Immunity in Infection, Malignancy and Autoimmunity (NIIMA) Universal Scientific Education and Research Network (USERN) Tehran Iran; ^2^ Department of Medical Laboratory Sciences, School of Allied Medical Sciences Tehran University of Medical Sciences Tehran Iran; ^3^ Student Research Committee Shahid Beheshti University of Medical Sciences Tehran Iran; ^4^ Medical School Shahid Beheshti University of Medical Sciences Tehran Iran; ^5^ Research Center for Immunodeficiencies, Children's Medical Center Tehran University of Medical Sciences Tehran Iran; ^6^ Department of Immunology, School of Medicine Tehran University of Medical Sciences Tehran Iran

**Keywords:** computational neurobiology, gut‐brain axis, major depressive disorder, microbiome

## Abstract

**Background and Aims:**

Major Depressive Disorder (MDD) is a highly common neuropsychiatric disorder globally. A variety of factors contribute to the neuropathology of MDD. Microbiome research in neuropsychiatric disorders such as MDD has recently attracted attention. Indeed, the gut‐brain axis could influence the course of MDD through metabolites such as Gamma‐Aminobutyric Acid (GABA), Quinolinate, and other factors. Such metabolites may modulate the balance of excitatory and inhibitory signals. Moreover, MDD features abundant hyperinflammatory bacteria, whereas anti‐inflammatory butyrate‐synthesizing genera are decreased.

**Methods:**

Despite mounting evidence on the implications for the microbiome in MDD, it is unclear whether a bidirectional or causal relationship is in effect. To overcome this challenge, researchers have utilized AI tools to investigate the complex association between the microbiome and MDD.

**Results:**

Additionally, there is no solid biomarker recognized for diagnosis and prognosis of MDD, while further application of AI using ML protocols, such as random forest, NNs, SVM, and DL models, could offer a rather solid and reliable comprehension of the complicated nature of microbiome‐MDD interplay.

**Conclusions:**

The present article reviews microbiome alterations as well as inflammatory and metabolic pathways in MDD with a focus on AI technology including support vector machines (SVM), random forests (RF), deep neural networks (DNNs), and autoencoders, which are used to identify microbial biomarkers, predict treatment results, and support personalized medicine.

## 1 Introduction

Major Depressive Disorder (MDD) is among the most common neuropsychiatric disorders, influencing over 300 million individuals worldwide [1, 2]. Although multiple investigations have been carried out on the relationship between MDD and neurotransmitters, the neuropathology of MDD is not yet fully understood. Meanwhile, a myriad of factors could influence the course of MDD, including genetic, environmental, psychiatric, and neurobiological elements [3].

Among the non‐hereditary elements that contribute to MDD, the gut microbiome has garnered attention in recent years. In terms of correlating microbiota diversity parameters with clinical depression, numerous experiments suggested a connection between dysbiosis and depression. Previous studies suggested remarkable gut microbiome alterations in MDD patients, particularly in the diversity of microbiome as well as the approximate abundance of special bacterial taxa [4]. Meanwhile, recent studies showed the effects of the gut microbiota on the nervous system through modulation of metabolism in the gastrointestinal system or mediation of signaling mechanisms by various bacterial components [5].

The gut microbiota consists of numerous species of bacteria, with separate subspecies associated with special secretory and metabolic characteristics. Certain bacteria produce neurotransmitters; for example, *Escherichia* and *Enterococcus* may be responsible for serotonin synthesis, whereas some bacteria, such as Bifidobacterium or Lactobacillus, synthesize Gamma‐Aminobutyric Acid (GABA). The microbiome could also trigger the central nervous system (CNS) through the synthesis of bacterial metabolites [6]. On the other hand, in MDD, dysregulation of the hypothalamic‐pituitary‐adrenal (HPA) axis and molecular immunoinflammatory and oxidative stress alterations as well as changed tone of the vagal nerve have been suggested to play a role [7] as well as the disproportion of excitatory‐inhibitory processes [8, 9]. Changes in microbiome diversity and altered abundance of specific taxa among gut microbiota, which are found in MDD cases, may be related to its core neuropathogenesis. Nevertheless, a definitive causal relationship between the two has not been proven. MDD is linked to microbial dysbiosis, which is explained as an alteration of microbiome diversity as a result of interrupted composition rates of microbiota along with associated functional alterations [10, 11, 12, 13].

Although gut microbiota seems to have a key role in MDD neuropathogenesis, challenges remain, and the definitive contribution of dysbiosis remains vague. Meanwhile, whether microbial dysbiosis is in a cause‐and‐effect association with the brain or solely a result of MDD‐associated neuropathological alterations is a challenge [4]. There are also some other challenges in the diagnosis of MDD [14].

With the rapid progress in Artificial Intelligence (AI) tools, researchers have utilized the power of AI to decipher the relationship between the microbiome and MDD [15]. Following appropriate training, the ability to analyze large‐scale data and algorithms could be utilized for tasks ranging from basic medical diagnosis tools to much more complicated analyses such as omics [16, 17]. Microbiome metabolites and their molecular effects could form crossroads with a variety of molecules involved in such pathways associated with MDD neuropathology, forming a complex interplay [2]. AI‐based microbiome analysis has recently been used to distinguish MDD from other neuropsychiatric disorders and/or healthy individuals [18].

In the present review, we discuss microbiota alterations, microbiome inflammatory and metabolic pathways in MDD, and the utility of AI technology in deciphering the complex microbiome‐depression interplay. Finally, a new perspective is provided to improve upcoming research attempting to develop microbiome‐based diagnostics for Major Depressive Disorder.

## 2 Overview of MDD

### 2.1 MDD Pathogenesis

Clinically, MDD could feature consistent low mood, loss of interest, fatigue, weight and appetite changes, sleep problems, impaired cognition, along with emotional symptoms such as guilt [3, 19]. The disorder also significantly increases the risk of suicide [20, 21].

The pathogenesis of MDD is complex and is associated with a combination of genetic, environmental, and neurobiological factors [22]. Disproportionate excitatory‐inhibitory neurotransmitter balance, impaired neural plasticity, and dysfunctions in stress response systems contribute to the disorder [21]. The monoamine view of depression indicates that deficiencies in serotonin (5‐HT), dopamine (DA), or norepinephrine (NE) contribute to the development of depression [23]. Altered glutamatergic and GABAergic systems are linked to depression, representing an imbalance of excitatory‐inhibitory neurotransmission. Augmented NMDA receptor activity, attenuated glutamate transporters, and interrupted synaptic plasticity activate excitatory mechanisms, whereas decreased GABA and receptor changes impair inhibitory control, contributing to MDD. This imbalance could offer novel therapeutic and diagnostic targets; however, additional studies are required [24, 25].

Moreover, a neuroendocrine‐immune mechanism is involved in depression. Stress induces microglia, as a result, influencing the HPA axis and corticosteroid production, generating a cycle that exacerbates depression. Additionally, tryptophan (TRP) metabolism is changed, with abundant generation of neurotoxic metabolites including quinolinic acid (QUIN) that influences neurotransmitters, especially excitatory transmitters [20].

The neuropathology of MDD goes beyond neurotransmitter imbalances; it is appropriate to view clinical MDD as a result of complex interactions of neurogenetics, neurophysiological, and neuroimmunological factors, along with external stress [26].

### 2.2 Epigenetics

Epigenetic changes could comprise DNA methylation, modifications of histone, as well as acetylation [27]. It has garnered significant attention, leading to large‐scale epigenomic studies [28]. It seems that a wide range of diseases, including MDD, are influenced by dysregulated epigenetics [29, 30, 31]. There is evidence demonstrating epigenetic modifications taking place early in life and driving lifelong susceptibility to being stressed [32]. The study by Weaver et al. [33] in rodents is a good example, while offspring of mothers that received lower amounts of maternal care or grooming (LG) indicated enhanced stress reactivity as well as anxiety‐associated neurobehavior in their adult life, in comparison to the ones experiencing high maternal care or grooming (HG). In the low‐maternal care or grooming group of rodents, researchers found attenuated hippocampal glucocorticoid receptor (GR) expression together with reduced acetylation of H3K9 and enhanced DNA methylation close to the GR promoter in comparison to HG animals. These alterations may contribute to difference in stress reactivity and neurobehavioral outcomes associated with low‐ versus high‐LG maternal care [33]. Results from this work indicate that epigenetic regulation of certain additional genes could have been involved. This response to stress preservation could as a result be neurobehaviorally transmitted without necessarily representing actual epigenetic hereditament [34].

### 2.3 Non‐Coding RNAs

Non‐coding RNAs (ncRNAs) denote a heterogeneous class of transcriptions which that do not result in functional proteins. Ever since they were discovered, ncRNAs have surfaced as key mediators of several roles in various cellular processes. ncRNA upregulation and downregulation could be associated with a signature linked to disease [35]. Of note, there are multiple experiments revolving around the linkage among microRNAs (miRNAs) and disorders in humans [36]. Meanwhile, certain groups of ncRNAs, like long non‐coding RNAs (lncRNAs) and circular RNAs (circRNAs), have been suggested to play associative roles in psychiatric disorders, including yet not limited to MDD [37, 38].

LncRNAs could potentially contribute to the neuropathogenesis of MDD via a variety of pathways, including regulating neurotransmitters and neurotrophic factors, influencing synaptic conduction, and modulating the ventriculo‐olfactory neurogenic system [37]. In addition, depending on genome‐wide association studies (GWAS) as well as molecular investigations, the implications of lncRNA as a plausible marker for the precise diagnosis of MDD or other psychiatric disorders could be better defined. As a result, it is crucial to investigate if lncRNAs could act as plausible treatments targets and diagnostic markers for MDD. ncRNAs could function as competitive endogenous RNAs (ceRNAs) that influence miRNAs via response factors [39], as a result influencing gene expression. Gut microbiome balance could be associated with changes to the expression of the hippocampal LncRNA‐miRNA‐mRNA axis, which could indicate a regulatory role for gut microbiota in post‐transcriptional gene expression. These influences could potentially be implicated in MDD [40]. Furthermore, researchers established an lncRNA‐miRNA‐mRNA‐pathway‐drug network of depression via computational approaches to broaden knowledge of the implications of lncRNA in MDD, warranting further epigenetics study in depressive disorders [41].

### 2.4 Microbiome

Microbiota partakes in activation, training, and proper functionality of the human immunity. Additionally, the immune system has undergone evolution as a way to sustain the symbiotic relation of the host with greatly diverse and changing microbes [42].

MDD has increasingly been linked to systemic alterations in both peripheral blood and gut microbiome. However, the exact mechanisms remain unclear [26]. The gut‐brain axis could have a major role in connecting gut health with MDD, as gut immune responses can elevate pro‐inflammatory cytokines [43].

## 3 MDD‐Related Microbiota

The dominant gut microbiota mainly comprises *bacteria* particularly *Firmicutes*, and their natural presence is essential for the functioning of a healthy body [44]. Accumulating evidence has revealed that gut microbiota could modulate intestinal neurotransmitter metabolism, thereby affecting the gut‐brain axis (GBA) and influence the functioning of the CNS, which can play a key role in the development of psychological diseases such as MDD [26]. The imbalance in the gut microbiome can manifest in various ways, for example, high abundance of *Streptococcus*, low abundance of *Lactobacillus*, and can be detected using the 16S ribosomal RNA (rRNA) sequencing approach [26].

There are clear differences in immune cells, metabolic activities, and the gut microbiome between individuals with MDD and healthy individuals. Based on reports, MDD cases showed higher levels of *Bacteroidaceae*, *Desulfovibrionaceae*, and *Enterococcaceae*, while healthy controls showed higher levels of Firmicutes, *Erysipelatoclostridiaceae*, and *Monoglobaceae* [26, 45]. Indeed, MDD patients showed higher levels of memory B cells and CCR2 + CD8 T cells [26]. Additionally, gut microbiota composition is also altered in MDD patients. Changes in *Lactobacillus* species influence immune cell activity, while microbial metabolites like short‐chain fatty acids (SCFAs) regulate immune and neural functions, suppress neuroinflammation, and modulate mood as they can permeate the blood–brain barrier (BBB). Additionally, serotonin (5‐HT) and GABA production could be affected, further exacerbating depression through vagus nerve activation [46, 47, 48].

Gut hormones, including Glucagon‐like peptide‐1 (GLP‐1) and Cholecystokinin (CCK), influence cognition and emotions, with GLP‐1 receptor agonists showing potential in reducing neuroinflammation and improving mood [49]. MDD itself is associated with altered gut microbiota composition, increasing inflammatory‐promoting bacteria such as *Bacteroidaceae*, *Desulfovibrionaceae*, and *Enterococcaceae*. This bidirectional relationship suggests that targeting gut microbiota, inflammation, and microbial metabolites may offer new diagnostic approaches [50].

## 4 Microbiome Biomarkers in MDD

Studies on microbiome alterations in MDD led to several potential biomarkers [51, 52]. Microbial genes and fecal metabolites, including metabolic microbiome biomarkers and inflammatory factors in serum, can significantly contribute to the process of diagnosis and treatment of MDD [51, 52].

Altered GABA synthesis was reported and factors involved in the glutamine‐to‐GABA, including glsA, gltB, and GLT were also changed [52]. On the other hand, BetB, as a microbial enzyme‐related gene that contributes to arginine metabolism, is downregulated in MDD. Also, AOC3, hpaB, hpaE, hpaG, and homovanillate, connected with the phenylalanine metabolic pathways, were decreased. Moreover, the KYNU gene, which is involved in kynurenine pathway metabolism, was upregulated in patients with MDD [52].

In a study exploring the composition of the gut microbiome in MDD patients, N‐acetylornithine, proline, oxoproline, and glutathione, which are related to GABA increase, and stool quinolinate levels decreased. Also, fecal glycerophospholipid seems to have an essential correlation between microbiota and depression [10, 53].

It is worth mentioning that some other factors can be considered MDD biomarkers, whose increases or decreases in serum due to microbiome changes can be tracked [54]. Using Random Forest, a Machine Learning algorithm generally applied for classification and regression tasks, the study introduced Taurocholic acid, Docosahexaenoic acid, and LysoPC levels in serum samples as noteworthy inflammatory biomarkers differentiating MDD patients from healthy controls [51]. In another study, a number of Operational Taxonomic Units (OTUs), bacteria including Firmicutes as well as Apolipoprotein A1 (APOA1), Alpha‐1 Antitrypsin (AAT), Neutrophil Percentage (Neu%), and Basophil (Baso), were introduced as MDD biomarkers, using a logistic regression model [54]. In another study, gut microbiome‐related inflammatory serum metabolites, as biomarkers for depression, were investigated. Total Protein (TP), Globulin (GLB), Eosinophil Percentage (EO%), APN, Apolipoprotein B (APOB), APOA1, Total Cholesterol (TC), Alpha‐1 Antitrypsin (AAT), Albumin (ALB), Lymphocyte Percentage (Lym%), pre‐albumin (PAL B), RBC, C‐Reactive Protein (CRPHS), Neu%, and Baso% were introduced as inflammation factors, using random forest; NEUT% and BASO are upregulated in MDD patients, while TP, GLB, EO%, APN, TC, APOA1, APOB, and AAT are downregulated [51, 54]. When compared, it appears that the second one has a more targeted approach, introducing differential biomarkers.

## 5 Machine Learning for Predicting MDD Using Microbiome Data: Unleashing the Power of Artificial Intelligence

### 5.1 Limitations of Biomarkers in Traditional Diagnosis of MDD

Standard diagnosis of MDD is only reliable clinically through history taking and psychiatric evaluation. This approach is susceptible to false positives and false negatives in diagnosis. Additionally, precision medicine in MDD pharmacotherapy has not been optimally successful [52]. The discovery of new tools such as high‐precision sequencing, computational biology, and different Machine Learning (ML) or Artificial Intelligence (AI) algorithms offers new opportunities for current studies to understand the complicated relationship between microbiome and host's nervous system [55].

### 5.2 Next‐Generation Approaches to Computational Microbiome‐Based MDD Diagnosis

In the past decade, ML‐based approaches could enable efficient processing, categorization, and identification of interlinks between microbiome and MDD. ML algorithms, which may be useful to study the microbiome‐MDD relationship, comprise logistic regression, Random Forest (RF), support vector machine (SVM), k‐nearest neighbor (KNN), Artificial Neural Networks (ANN), and Deep Learning (DL) [56, 57].

**Figure 1:**
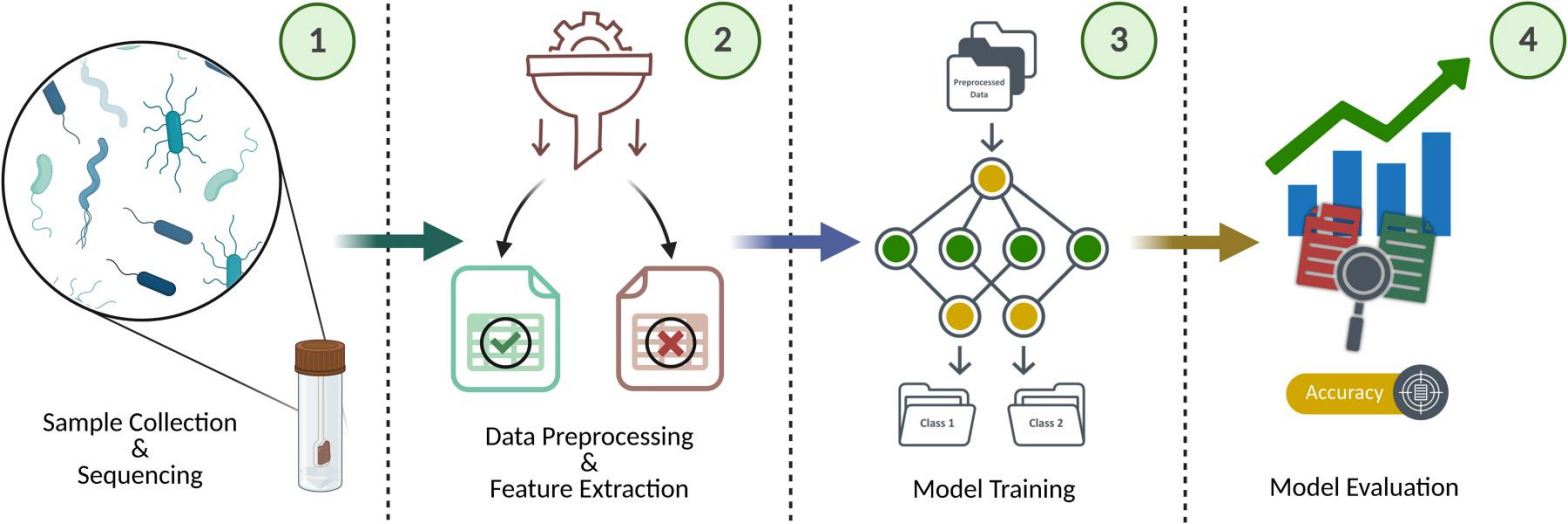
Overview of AI‐based steps for diagnosing MDD using microbiome data.

Figure 1 shows the overall process of AI‐driven diagnosis of MDD using microbiome analysis. A common Machine Learning pipeline begins by training the model on a subset of features using a training dataset. Model performance can be evaluated on a separate validation set during tuning, and then it can be tested on an independent test set to assess generalization. It is necessary to consider data preprocessing steps to ensure robustness. These steps include appropriate feature selection, avoidance of batch effects, noise reduction, and ensuring adequate sample size. Metagenomic data comprises a massive number of variables or “dimensions” that could be used to describe the sampled data. Dimensionality reduction may be quintessential to handle high‐dimensional omics data. This could be achieved using biostatistical techniques, such as principal component analysis (PCA). These key features could be the basis of data categorization and segregation of different classes or forecasting variables [2].

Omics datasets are inherently complex and involve an unmanageable number of factors, which render manual/traditional analyses unfruitful. Through the application of data‐training protocols, the large number of parameters could be managed. Moreover, ML methods are generally categorized as either:

Supervised ML: the algorithm learns from labeled training datasets to make predictions for unseen test data;

Unsupervised ML: the algorithm does not involve labeling and classifies the dataset into groups or detects complex patterns [57].

In order to evaluate the efficiency or robustness of AI‐based technology, quantifiable facets and performance metrics are necessary. These comprise the following parameters:

i. Accuracy: Proportion of correctly predicted instances (both positives and negatives) out of all predictions.
ii. Precision: True positives divided by all predicted positives.
iii. Recall: True positives divided by all actual positives.
iv. F1‐Score: Harmonic mean of precision and recall.
v. Area Under the Receiver Operating Characteristic Curve (AUC‐ROC): Measures the model's ability to differentiate between classes for different thresholds by plotting the true positive rate against the false positive rate. Higher AUC means better separability.
vi. Mean squared error (MSE): The average of the squared differences between predicted and actual values.
vii. *R* ^2^ (Coefficient of Determination): Proportion of variance in the dependent variable that is explained through the model.
viii. Calibration curve: A plot that compares predicted probabilities from a classification model with actual observed outcome frequencies. It is used to evaluate how well predicted risks reflect true probabilities [58].

ML algorithms can identify alterations in the metagenomic signature that could characterize the microbiome of individuals with MDD, and can build a classifier of the cases' mental state according to genomic signature [56].

Feature selection in ML ranks the importance of each variable based on its effect on the model's predictive performance. Moreover, Omics datasets contain a variety of events, including single nucleotide variations (SNVs), differentially expressed genes (DEGs), or DNA methylation regions. ML may be valuable for the analysis of transcriptome, as conventional biostatistics often fails to fully take into account the fine genetic interactions present in the dataset. ML enables researchers to both detect special pathology‐associated genes and study non‐linear genetic associations [57]. Intriguingly, such patterns are able to accomplish several goals: classifying MDD or determining the severity of disease by clinical and paraclinical variables, supporting the development of personalized treatment strategies, predicting treatment response or therapeutic outcomes, and offering insights for personalized treatments through the analysis of gene profiles. Moreover, such approaches make possible the early identification of comorbid conditions like anxiety disorders, improving the therapeutic outlook. Further, ML could be implemented in prospective studies to track MDD cases to spot relapse and efficient resolution of clinical symptoms in relation to the microbiome profile, offering insights for timely intervention. ML could also classify MDD cases by risk, assisting precision prevention of complications such as suicide [58].

Omics data must first be preprocessed using normalization and data transformation to prepare it for input into deep neural networks (DNNs). DNNs are capable of modeling complex non‐linear interconnections, rendering them suitable for textual evaluation. DNNs can estimate probability distributions for the purpose of classification queries or numerical values for the purpose of regression analyses. Other approaches, such as batch normalization as well as hyperparameter tuning, could be utilized to improve the performance and stability when training. In the settings of MDD, the DNN protocol could be utilized to predict remission to individual antidepressants instead of classifying them as selective serotonin reuptake inhibitors (SSRIs) or serotonin‐norepinephrine reuptake inhibitors (SNRIs), which could be useful for personalization purposes. Hyperparameter tuning could improve model performance through systematic methods such as grid searching. In order to prevent overfitting, techniques such as dropout, batch normalization, early stopping, and cross‐validation can be employed. The ultimate goal of applying DNNs to the MDD–microbiome relationship is to evaluate therapeutic efficacy and enhance clinical practicality [58].

It is noteworthy that AI could assist the therapy of gut disorders and NLRP3 inflammasome activation in psychiatric disorders, including MDD. In a general context, a recent study by Malwe and colleagues suggested the use of an ML model that used prior research on drug‐gut interactions to determine the effectiveness of experimental pharmacotherapeutics [59].

Machine Learning models such as Random Forest can be used to personalize the selection and dosage of prebiotics and probiotics for individual patients based on their microbiome profiles. While currently studied in other contexts, these approaches could hypothetically be used to select the most efficient microbiome strains and doses for recovering gut balance and treating MDD [55].

Logistic regression is a generalized linear approach which could have applications in binary classification problems. The Random Forest approach is highly efficient in microbiome community experiments. The SVM could be utilized in classification tasks of metagenomic data [60]. Nonetheless, some studies have studied SVM application to microbiota datasets because of their high dimensionality and noise sensitivity [61]. For this purpose, methods like the Least Absolute Shrinkage and Selection Operator (LASSO) or Elastic Net are applied more often. In recent years, various methods of DL have been utilized in metagenomic studies, such as ANNs for categorization and the building of models for forecasting. For instance, the proposed neural network was used as a classification model in a program for the detection of the disorder [62]. The tool was applied to both 16S rRNA data and whole metagenome sequencing datasets. You Only Look Once (YOLO) is another deep learning algorithm which could be applied to image classification tasks [63]. It is a state‐of‐the‐art object detection approach whose goal is to predict the category of an object and the bounding box that determines the object location on the input image [56].

Autoencoders, a kind of ANN, are useful for carrying out non‐linear dimension reduction through learning efficient coding of imported datasets. Such networks are efficient for complicated, nonlinear associations in microbiome data, rendering them optimal for isolation of features [64, 65]. Some applications indicate the versatility and efficiency of autoencoders in extracting useful information from complicated data [55].

### 5.3 AI‐Powered Studies Implementing ML Tools to Investigate the Relationship Between Microbiome and MDD

A study indicated that the alpha diversity of the intestine flora was not markedly different in MDD in comparison with healthy populations. However, the beta diversity was markedly changed. Machine Learning models identified a number of MDD‐exclusive bacterial markers, with *Alistipes*, *Dysosmobacter*, *Actinomyces*, *Ruthenibacterium*, as well as *Thomasclavelia* upregulated, whereas *Faecalibacterium*, *Pseudobutyrivibrio*, along with *Roseburia* were markedly reduced in MDD cases [66, 67].

Because genomic signatures could be favorable when represented in the form of heat maps, the image categorization approach using YOLO was selected in one of the studies. It demonstrated the implementation of the YOLO model in genomic experiments, in particular for the categorization of genomic signatures correlated with MDD [56].

Another experiment utilized five ML algorithms to detect genetic markers, including Bayesian Networks, Support Vector Machines, Random Forest, Backpropagation Neural Networks, and Linear Discriminant Analysis. The microbiome research used a random forest approach to detect microbiome taxa and associated metabolism related to MDD. Segregation of MDD and healthy controls was achieved. In the experiment of microRNAs, a regularized gradient boosted approach was utilized to categorize cases with MDD and control individuals. The models were trained with cross‐validation and 2500 iterative parameter searches. The built models were next re‐trained by the optimal parameters [57].

In one of the studies, the proposed framework utilized LASSO‐Penalized Regression to identify potential biologically meaningful relationships among genes and gut microbiome. Using this approach, they aimed to investigate intricate relationships between plasma metabolites and the microbiome, which could contribute to understanding the neurobiological underpinnings of MDD presentations [13]. The lasso regression utilizes shrinkage to carry out feature selection, retaining only a few taxa associated with a plasma metabolite. Through this approach, researchers found 169 meaningful plasma metabolite‐taxa relations in controls, whereas 680 associations were found in MDD patients. Intriguingly, there were no common relationships among the controls and MDD cases, with only six metabolites found related to certain microbiome taxa in both subgroups. In addition, the analysis showed a meaningful change in the alteration of circulating metabolites could be explained via microbiome changes in MDD and healthy individuals. However, a greater proportion of variance was explainable in the MDD subgroup. Such findings indicate that microbiome has a more potent influence on circulating metabolites in MDD cases. Overall, MDD‐specific algorithms are identified in the relationship of circulating metabolites and microbiome [58].

Wang et al. used an SVM Recursive Feature Elimination (RFE) approach, which is an SVM model with improved variable selection in a predictive model by reducing the number of feature vectors. The SVM‐RFE protocol detected eight bacterial communities with meaningful changes among phyla/genera. The study showed *Alistipes*, *Dysosmobacter*, *Actinomyces*, *Ruthenibacterium*, and *Thomasclavelia* were increased, whereas Faecalibacterium, Pseudobutyrivibrio, along with Roseburia were decreased. These bacterial taxa could serve as candidate markers for MDD [66].

Several studies have attempted to classify MDD cases based on functional features of the gut microbiota using models such as random forest, Elastic Net, and YOLO. To achieve this aim, data from the Whole Metagenome Sequencing were used. In their study, Angelova et al. classified samples in MDD and healthy controls and showed YOLO could effectively analyze metagenomic samples and corroborate a reduction in the amount of *Faecalibacterium prausnitzii* for the manifestation of MDD [56].

Overall, by using Machine Learning algorithms such as SVM and RF, advanced model architectures like CNNs and RNNs that can be used for pattern recognition, and dimensionality reduction techniques including PCA and autoencoders, scientists can decipher complex microbiome characteristics [55].

## 6 Discussion

The growing body of literature on the microbiota–gut–brain axis (MGBA) introduces the gut microbiome as a potential master regulator of neurophysiological and behavioral processes. Dysbiosis has been associated with gastrointestinal disorders and neuropsychiatric diseases, such as anxiety, depression, and neurodevelopmental disorders. The findings suggest that microbial signal molecules and metabolites are able to affect brain function through complex mechanisms such as immune modulation, vagus nerve transmission, and neurotransmitter synthesis. Despite the causal associations that have been generated using preclinical models, the results have yet to be successfully translated into the creation of efficacious human treatments. Heterogeneity of the individual microbiome, environmental influences, and methodological discrepancies between investigations limit the translation of extant findings. However, promising accounts of microbiome‐targeted interventions, that is, probiotic supplementations, diet alteration, and fecal microbiota transplantation, suggest that manipulation of the gut microbiota through therapy can emerge as a future adjuvant treatment strategy for CNS disorders. Interdisciplinary research efforts are needed to clarify the mechanisms of gut‐brain interaction and to develop standardized procedures for clinical practice [68, 69, 70].

There are also some limitations. Existing studies use observational or cross‐sectional designs. This restricts the ability to establish a relationship between changes in the gut microbiome and MDD. Microbiome data can also differ because of variations in sequencing technologies, population diversity, diet, and health conditions, which also pose challenges regarding generalizability. Machine Learning models have good potential for identifying patterns. However, many of them are experimental and cannot be used in real‐world clinical environments. AI methods can demonstrate statistical robustness, but model transparency and interpretability can still pose challenges in deep learning approaches.

Advancing this field requires more research on longitudinal and interventional designs to help understand causal mechanisms. It is necessary to align data preprocessing workflows, standardize evaluation metrics for Machine Learning algorithms, and use open‐access datasets to improve the reliability and robustness of computational models. A better understanding of the disorder can also be achieved by integrating various omics layers, such as transcriptomic, proteomic, and metabolomic data. Another important aspect is how personal factors like diet, medications, and genetic predisposition can interact with the gut‐brain axis to affect depressive symptoms and help identify more personalized treatments.

## 7 Conclusions

Evidence shows that specific taxa are altered in MDD. The relationship between microbiome and MDD could be explained by metabolites or bacterial components secreted by the microbiome, changes to absorption and metabolism, or the release of inflammatory factors. However, the complex nature of their relationship may prompt researchers to take on computational ML‐based methods. Traditional approaches have not yielded useful diagnostic biomarkers for MDD. On the contrary, ML protocols such as random forest, SVMs, NNs, and DL models may be able to unravel high‐dimensional data, which are characteristic of metagenomic experiments common in the field of microbiome. Such technology may facilitate important procedures, including feature selection, dimensionality reduction, and pattern detection to provide useful biomarkers to differentiate MDD, predict the clinical course of MDD, or determine response to antidepressant treatments. Additionally, employing image classification methods to analyze metagenomic heat maps indicates the versatility of AI technologies in decoding the microbiome‐MDD relationship. Current challenges of AI tools include data noise, class imbalance, and limited sample sizes. Overall, while no consensus diagnostic or prognostic marker for MDD has been suggested, extended utilization of AI in future studies is warranted to draw more reliable conclusions on the complex nature of microbiome‐MDD interplay.

## Author Contributions

**Nazanin Zahra Keshvari:** conceptualization, writing – original draft, writing – review and editing, methodology, project administration, formal analysis. **Sara Asl Motaleb Nejad Sarkhab:** conceptualization, writing – original draft, writing – review and editing. **Tara Shahmoradi:** conceptualization, writing – original draft, writing – review and editing, visualization. **Mohammad Pourashory:** conceptualization, writing – original draft, writing – review and editing, data curation, investigation. **Arash Esmaeili:** conceptualization, writing – original draft, writing – review and editing. **Kiarash Saleki:** conceptualization, writing – review and editing, writing – original draft, supervision, project administration, formal analysis. **Nima Rezaei:** conceptualization, supervision, writing – original draft, writing – review and editing, project administration, formal analysis, funding acquisition.

## Ethics Statement

The authors have nothing to report.

## Conflicts of Interest

The authors declare no conflicts of interest.

## Transparency Statement

The corresponding author, Nima Rezaei, on behalf of all co‐authors, affirms that this manuscript is an honest, accurate, and transparent account of the study being reported; that no important aspects of the study have been omitted; and that any discrepancies from the study as planned (and, if relevant, registered) have been explained.

## Data Availability

Data sharing is not applicable to this article as no datasets were generated or analyzed during the current study.

## References

[1] F. Guo , L. Jing , Y. Xu , et al., “Gut Microbiota and Inflammatory Factor Characteristics in Major Depressive Disorder Patients With Anorexia,” BMC Psychiatry 24, no. 1 (2024): 334. 38698338 10.1186/s12888-024-05778-0 PMC11067108

[2] O. V. Averina , E. U. Poluektova , Y. A. Zorkina , A. S. Kovtun , and V. N. Danilenko , “Human Gut Microbiota for Diagnosis and Treatment of Depression,” International Journal of Molecular Sciences 25, no. 11 (2024): 5782. 38891970 10.3390/ijms25115782 PMC11171505

[3] M. Fakhoury , “New Insights into the Neurobiological Mechanisms of Major Depressive Disorders,” General Hospital Psychiatry 37, no. 2 (2015): 172–177. 25772946 10.1016/j.genhosppsych.2015.01.005

[4] L. Liu , H. Wang , X. Chen , Y. Zhang , H. Zhang , and P. Xie , “Gut Microbiota and Its Metabolites in Depression: From Pathogenesis to Treatment,” EBioMedicine 90 (2023): 104527. 36963238 10.1016/j.ebiom.2023.104527 PMC10051028

[5] K. Saleki , P. Alijanizadeh , N. Javanmehr , and N. Rezaei , “The Role of Toll‐Like Receptors in Neuropsychiatric Disorders: Immunopathology, Treatment, and Management,” Medicinal Research Reviews 44, no. 3 (2024): 1267–1325. 38226452 10.1002/med.22012

[6] V. M. Lowe , M. Chaplin , and D. Sgambato , “Major Depressive Disorder and the Gut Microbiome: What Is the Link?,” General Psychiatry 36, no. 1 (2023): e100973. 36844965 10.1136/gpsych-2022-100973 PMC9943825

[7] C. A. Köhler , T. H. Freitas , M. Maes , et al., “Peripheral Cytokine and Chemokine Alterations in Depression: A Meta‐Analysis of 82 Studies,” Acta Psychiatrica Scandinavica 135, no. 5 (2017): 373–387. 28122130 10.1111/acps.12698

[8] A. S. Chambers and J. J. B. Allen , “Vagal Tone as an Indicator of Treatment Response in Major Depression,” Psychophysiology 39, no. 6 (2002): 861–864. 12462513 10.1111/1469-8986.3960861

[9] S. Ghosal , B. D. Hare , and R. S. Duman , “Prefrontal Cortex Gabaergic Deficits and Circuit Dysfunction in the Pathophysiology and Treatment of Chronic Stress and Depression,” Current Opinion in Behavioral Sciences 14 (2017): 1–8. 27812532 10.1016/j.cobeha.2016.09.012 PMC5086803

[10] Q. Zhong , J. Chen , Y. Wang , W. Shao , C. Zhou , and P. Xie , “Differential Gut Microbiota Compositions Related With the Severity of Major Depressive Disorder,” Frontiers in Cellular and Infection Microbiology 12 (2022): 907239. 35899051 10.3389/fcimb.2022.907239 PMC9309346

[11] F. Guo , L. Jing , Y. Xu , K. Zhang , H. Zhang , and P. Liu , “Characteristics of Intestinal Flora in Patients With Major Depressive Disorder and Its Correlation With Inflammatory Factors,” Chinese Journal of Microecology 36, no. 7 (2024): 788–795.

[12] J. K. Knudsen , C. Bundgaard‐Nielsen , S. Hjerrild , R. E. Nielsen , P. Leutscher , and S. Sørensen , “Gut Microbiota Variations in Patients Diagnosed With Major Depressive Disorder—A Systematic Review,” Brain and Behavior 11, no. 7 (2021): e02177. 34047485 10.1002/brb3.2177 PMC8323045

[13] Y. Wang , J. Zhou , J. Ye , et al., “Multi‐Omics Reveal Microbial Determinants Impacting the Treatment Outcome of Antidepressants in Major Depressive Disorder,” Microbiome 11, no. 1 (2023): 195. 37641148 10.1186/s40168-023-01635-6 PMC10464022

[14] A. Berry , M. Luciano , F. Cirulli , and A. Fiorillo , “The Future of Diagnosis in Mental Health: Promises and Challenges of Biomarkers to Identify Reliable and Highly Predictive Biosignatures of Affective Disorders,” European Psychiatry 68, no. 1 (2025): e32. 39911061 10.1192/j.eurpsy.2025.15 PMC11883774

[15] J. Mehltretter , C. Rollins , D. Benrimoh , et al., “Analysis of Features Selected by a Deep Learning Model for Differential Treatment Selection in Depression,” Frontiers in Artificial Intelligence 2 (2020): 31. 33733120 10.3389/frai.2019.00031 PMC7861264

[16] F. Stolfi , H. Abreu , R. Sinella , et al., “Omics Approaches Open New Horizons in Major Depressive Disorder: From Biomarkers to Precision Medicine,” Frontiers in Psychiatry 15 (2024): 1422939. 38938457 10.3389/fpsyt.2024.1422939 PMC11210496

[17] P. Hamet and J. Tremblay , “Artificial Intelligence in Medicine,” Metabolism: Clinical and Experimental 69 (2017): S36–S40. 10.1016/j.metabol.2017.01.011 28126242

[18] M. Yu , H. Jia , C. Zhou , et al., “Variations in Gut Microbiota and Fecal Metabolic Phenotype Associated With Depression by 16S rRNA Gene Sequencing and LC/MS‐Based Metabolomics,” Journal of Pharmaceutical and Biomedical Analysis 138 (2017): 231–239. 28219800 10.1016/j.jpba.2017.02.008

[19] K. S. Kendler , “The Phenomenology of Major Depression and the Representativeness and Nature of DSM Criteria,” American Journal of Psychiatry 173, no. 8 (2016): 771–780. 27138588 10.1176/appi.ajp.2016.15121509

[20] L. Cui , S. Li , S. Wang , et al., “Major Depressive Disorder: Hypothesis, Mechanism, Prevention and Treatment,” Signal Transduction and Targeted Therapy 9, no. 1 (2024): 30. 38331979 10.1038/s41392-024-01738-y PMC10853571

[21] M. Fouad , M. Tadros , and H. Michel , “A Comprehensive Review of the Pathophysiology of Depression,” Archives of Pharmaceutical Sciences Ain Shams University 8 (2024): 122–132.

[22] M. Aan Het Rot , S. J. Mathew , and D. S. Charney , “Neurobiological Mechanisms in Major Depressive Disorder,” Canadian Medical Association Journal 180, no. 3 (2009): 305–313. 19188629 10.1503/cmaj.080697 PMC2630359

[23] M. Hamon and P. Blier , “Monoamine Neurocircuitry in Depression and Strategies for New Treatments,” Progress in Neuro‐Psychopharmacology and Biological Psychiatry 45 (2013): 54–63. 23602950 10.1016/j.pnpbp.2013.04.009

[24] C. Pittenger , G. Sanacora , and J. H. Krystal , “The NMDA Receptor as a Therapeutic Target in Major Depressive Disorder,” CNS & Neurological Disorders Drug Targets 6, no. 2 (2007): 101–115. 17430148 10.2174/187152707780363267

[25] R. Maqsood and T. W. Stone , “The Gut‐Brain Axis, BDNF, NMDA and CNS Disorders,” Neurochemical Research 41 (2016): 2819–2835. 27553784 10.1007/s11064-016-2039-1

[26] Z. Xie , J. Huang , G. Sun , et al., “Integrated Multi‐Omics Analysis Reveals Gut Microbiota Dysbiosis and Systemic Disturbance in Major Depressive Disorder,” Psychiatry Research 334 (2024): 115804. 38417224 10.1016/j.psychres.2024.115804

[27] W. Dai , X. Qiao , Y. Fang , et al., “Epigenetics‐Targeted Drugs: Current Paradigms and Future Challenges,” Signal Transduction and Targeted Therapy 9, no. 1 (2024): 332. 39592582 10.1038/s41392-024-02039-0 PMC11627502

[28] A. Portela and M. Esteller , “Epigenetic Modifications and Human Disease,” Nature Biotechnology 28, no. 10 (2010): 1057–1068. 10.1038/nbt.1685 20944598

[29] C. Park , J. D. Rosenblat , E. Brietzke , et al., “Stress, Epigenetics and Depression: A Systematic Review,” Neuroscience and Biobehavioral Reviews 102 (2019): 139–152. 31005627 10.1016/j.neubiorev.2019.04.010

[30] N. Begum , A. Mandhare , K. P. Tryphena , et al., “Epigenetics in Depression and Gut‐Brain Axis: A Molecular Crosstalk,” Frontiers in Aging Neuroscience 14 (2022): 1048333. 36583185 10.3389/fnagi.2022.1048333 PMC9794020

[31] I. Šalamon Arčan , K. Kouter , and A. Videtič Paska , “Depressive Disorder and Antidepressants From an Epigenetic Point of View,” World Journal of Psychiatry 12, no. 9 (2022): 1150–1168. 36186508 10.5498/wjp.v12.i9.1150 PMC9521527

[32] Z. Cheng , J. Su , K. Zhang , H. Jiang , and B. Li , “Epigenetic Mechanism of Early Life Stress‐Induced Depression: Focus on the Neurotransmitter Systems,” Frontiers in Cell and Developmental Biology 10 (2022): 929732. 35865627 10.3389/fcell.2022.929732 PMC9294154

[33] I. C. G. Weaver , N. Cervoni , F. A. Champagne , et al., “Epigenetic Programming by Maternal Behavior,” Nature Neuroscience 7, no. 8 (2004): 847–854. 15220929 10.1038/nn1276

[34] E. J. Nestler , “Epigenetic Mechanisms of Depression,” JAMA Psychiatry 71, no. 4 (2014): 454–456. 24499927 10.1001/jamapsychiatry.2013.4291 PMC4057796

[35] M. Cheng , Y. Zhu , H. Yu , et al., “Non‐Coding RNA Notations, Regulations and Interactive Resources,” Functional & Integrative Genomics 24, no. 6 (2024): 217. 39557706 10.1007/s10142-024-01494-w

[36] K. Nemeth , R. Bayraktar , M. Ferracin , and G. A. Calin , “Non‐Coding RNAs in Disease: From Mechanisms to Therapeutics,” Nature Reviews Genetics 25, no. 3 (2024): 211–232. 10.1038/s41576-023-00662-1 37968332

[37] C. Prodan‐Bărbulescu , E. P. Şeclăman , V. Enătescu , et al., “Evaluating the Connection Between Micrornas and Long Non‐Coding Rnas for the Establishment of the Major Depressive Disorder Diagnosis,” Biomedicines 12, no. 3 (2024): 516. 38540129 10.3390/biomedicines12030516 PMC10968605

[38] B. Roy , A. K. Verma , Y. Funahashi , and Y. Dwivedi , “Deciphering the Epigenetic Role of Long Non‐Coding RNAs in Mood Disorders: Focus on Human Brain Studies,” Clinical and Translational Medicine 15, no. 3 (2025): e70135. 40038891 10.1002/ctm2.70135 PMC11879898

[39] L. Liu , H. Wang , X. Chen , et al., “Integrative Analysis of Long Non‐Coding RNAs, Messenger RNAs, and MicroRNAs Indicates the Neurodevelopmental Dysfunction in the Hippocampus of Gut Microbiota‐Dysbiosis Mice,” Frontiers in Molecular Neuroscience 14 (2021): 745437. 35087377 10.3389/fnmol.2021.745437 PMC8787131

[40] C. A. Simpson , C. Diaz‐Arteche , D. Eliby , O. S. Schwartz , J. G. Simmons , and C. S. M. Cowan , “The Gut Microbiota in Anxiety and Depression—A Systematic Review,” Clinical Psychology Review 83 (2021): 101943. 33271426 10.1016/j.cpr.2020.101943

[41] W.‐Z. Hao , Q. Chen , L. Wang , et al., “Emerging Roles of Long Non‐Coding RNA in Depression,” Progress in Neuro‐Psychopharmacology and Biological Psychiatry 115 (2022): 110515. 35077841 10.1016/j.pnpbp.2022.110515

[42] Y. Belkaid and T. W. Hand , “Role of the Microbiota in Immunity and Inflammation,” Cell 157, no. 1 (2014): 121–141. 24679531 10.1016/j.cell.2014.03.011 PMC4056765

[43] W. Jiao , J. Lin , Y. Deng , et al., “The Immunological Perspective of Major Depressive Disorder: Unveiling the Interactions Between Central and Peripheral Immune Mechanisms,” Journal of Neuroinflammation 22, no. 1 (2025): 10. 39828676 10.1186/s12974-024-03312-3 PMC11743025

[44] N. Yazdanpanah and N. Rezaei , “Introduction to Psychoneuroimmunology,” in PsychoNeuroImmunology: Volume 1: Integration of Psychology, Neurology, and Immunology (Springer, 2024), 1–16.

[45] S. J. Rhee , H. Kim , Y. Lee , et al., “The Association Between Serum Microbial DNA Composition and Symptoms of Depression and Anxiety in Mood Disorders,” Scientific Reports 11, no. 1 (2021): 13987. 34234173 10.1038/s41598-021-93112-z PMC8263754

[46] J. Cheng , H. Hu , Y. Ju , et al., “Gut Microbiota‐Derived Short‐Chain Fatty Acids and Depression: Deep Insight Into Biological Mechanisms and Potential Applications,” General Psychiatry 37, no. 1 (2024): e101374. 38390241 10.1136/gpsych-2023-101374 PMC10882305

[47] P. Strandwitz , K. H. Kim , D. Terekhova , et al., “Gaba‐Modulating Bacteria of the Human Gut Microbiota,” Nature Microbiology 4, no. 3 (2019): 396–403. 10.1038/s41564-018-0307-3 PMC6384127 30531975

[48] N. Israelyan , A. Del Colle , Z. Li , et al., “Effects of Serotonin and Slow‐Release 5‐Hydroxytryptophan on Gastrointestinal Motility in a Mouse Model of Depression,” Gastroenterology 157, no. 2 (2019): 507–521.e4. 31071306 10.1053/j.gastro.2019.04.022 PMC6650329

[49] J. Detka and K. Głombik , “Insights into a Possible Role of Glucagon‐Like Peptide‐1 Receptor Agonists in the Treatment of Depression,” Pharmacological Reports 73, no. 4 (2021): 1020–1032. 34003475 10.1007/s43440-021-00274-8 PMC8413152

[50] Z. A. Barandouzi , A. R. Starkweather , W. A. Henderson , A. Gyamfi , and X. S. Cong , “Altered Composition of Gut Microbiota in Depression: A Systematic Review,” Frontiers in Psychiatry 11 (2020): 541. 32587537 10.3389/fpsyt.2020.00541 PMC7299157

[51] S. Bai , J. Xie , H. Bai , T. Tian , T. Zou , and J.‐J. Chen , “Gut Microbiota‐Derived Inflammation‐Related Serum Metabolites as Potential Biomarkers for Major Depressive Disorder,” Journal of Inflammation Research 14 (2021): 3755–3766. 34393496 10.2147/JIR.S324922 PMC8354734

[52] J. Yang , P. Zheng , Y. Li , et al., “Landscapes of Bacterial and Metabolic Signatures and Their Interaction in Major Depressive Disorders,” Science Advances 6, no. 49 (2020): eaba8555. 33268363 10.1126/sciadv.aba8555 PMC7710361

[53] X.‐Y. Tian , J.‐W. Xing , Q.‐Q. Zheng , and P.‐F. Gao , “919 Syrup Alleviates Postpartum Depression by Modulating the Structure and Metabolism of Gut Microbes and Affecting the Function of the Hippocampal Gaba/Glutamate System,” Frontiers in Cellular and Infection Microbiology 11 (2021): 694443. 34490139 10.3389/fcimb.2021.694443 PMC8417790

[54] S. Bai , H. Bai , D. Li , Q. Zhong , J. Xie , and J. Chen , “Gut Microbiota‐Related Inflammation Factors as a Potential Biomarker for Diagnosing Major Depressive Disorder,” Frontiers in Cellular and Infection Microbiology 12 (2022): 831186. 35372107 10.3389/fcimb.2022.831186 PMC8965553

[55] Y. Lv , Y. Xian , X. Lei , S. Xie , and B. Zhang , “The Role of the Microbiota‐Gut‐Brain Axis and Artificial Intelligence in Cognitive Health of Pediatric Obstructive Sleep Apnea: A Narrative Review,” Medicine 103, no. 50 (2024): e40900. 39686454 10.1097/MD.0000000000040900 PMC11651515

[56] I. Y. Angelova , A. S. Kovtun , O. V. Averina , T. A. Koshenko , and V. N. Danilenko , “Unveiling the Connection Between Microbiota and Depressive Disorder Through Machine Learning,” International Journal of Molecular Sciences 24, no. 22 (2023): 16459. 38003647 10.3390/ijms242216459 PMC10671666

[57] B. Taylor , M. Hobensack , S. Niño de Rivera , Y. Zhao , R. Masterson Creber , and K. Cato , “Identifying Depression Through Machine Learning Analysis of Omics Data: Scoping Review,” JMIR Nursing 7 (2024): e54810. 39028994 10.2196/54810 PMC11297379

[58] G. Corrivetti , F. Monaco , A. Vignapiano , et al., “Optimizing and Predicting Antidepressant Efficacy in Patients With Major Depressive Disorder Using Multi‐Omics Analysis and the Opade AI Prediction Tools,” Brain Sciences 14, no. 7 (2024): 658. 39061399 10.3390/brainsci14070658 PMC11275115

[59] A. S. Malwe and V. K. Sharma , “Application of Artificial Intelligence Approaches to Predict the Metabolism of Xenobiotic Molecules by Human Gut Microbiome,” Frontiers in Microbiology 14 (2023): 1254073. 38116528 10.3389/fmicb.2023.1254073 PMC10728657

[60] A. Statnikov , M. Henaff , V. Narendra , et al., “A Comprehensive Evaluation of Multicategory Classification Methods for Microbiomic Data,” Microbiome 1, no. 1 (2013): 11. 24456583 10.1186/2049-2618-1-11 PMC3960509

[61] R. Hernández Medina , S. Kutuzova , K. N. Nielsen , et al., “Machine Learning and Deep Learning Applications in Microbiome Research,” ISME Communications 2, no. 1 (2022): 98. 37938690 10.1038/s43705-022-00182-9 PMC9723725

[62] Y. Mreyoud , M. Song , J. Lim , and T. H. Ahn , “Megad: Deep Learning for Rapid and Accurate Disease Status Prediction of Metagenomic Samples,” Life 12, no. 5 (2022): 669. 35629336 10.3390/life12050669 PMC9143510

[63] T. Stark , V. Ştefan , M. Wurm , R. Spanier , H. Taubenböck , and T. M. Knight , “Yolo Object Detection Models Can Locate and Classify Broad Groups of Flower‐Visiting Arthropods in Images,” Scientific Reports 13, no. 1 (2023): 16364. 37773202 10.1038/s41598-023-43482-3 PMC10541899

[64] M. Ding , Y. Lang , H. Shu , J. Shao , and L. Cui , “Microbiota‐Gut‐Brain Axis and Epilepsy: A Review on Mechanisms and Potential Therapeutics,” Frontiers in Immunology 12 (2021): 742449. 34707612 10.3389/fimmu.2021.742449 PMC8542678

[65] A. Mhanna and Z. Alshehabi , “The Microbiota‐Gut‐Brain Axis and Three Common Neurological Disorders: A Mini‐Review,” Annals of Medicine & Surgery 85, no. 5 (2023): 1780–1783. 37228957 10.1097/MS9.0000000000000552 PMC10205337

[66] X. Wang , D. Cao , H. Zhang , W. Chen , J. Sun , and H. Hu , “Utilizing Metagenomic Profiling and Machine Learning Model to Identify Bacterial Biomarkers for Major Depressive Disorder,” Frontiers in Psychiatry 16 (2025): 1539596. 40041700 10.3389/fpsyt.2025.1539596 PMC11876151

[67] M. Gao , J. Wang , P. Liu , et al., “Gut Microbiota Composition in Depressive Disorder: A Systematic Review, Meta‐Analysis, and Meta‐Regression,” Translational Psychiatry 13, no. 1 (2023): 379. 38065935 10.1038/s41398-023-02670-5 PMC10709466

[68] V. Bamicha , P. Pergantis , and A. Drigas , “The Effect of Gut Microbiome, Neurotransmitters, and Digital Insights in Autism,” Applied Microbiology 4, no. 4 (2024): 1677–1701.

[69] S. G. Sorboni , H. S. Moghaddam , R. Jafarzadeh‐Esfehani , and S. Soleimanpour , “A Comprehensive Review on the Role of the Gut Microbiome in Human Neurological Disorders,” Clinical Microbiology Reviews 35, no. 1 (2022): e00338‐20. 34985325 10.1128/CMR.00338-20 PMC8729913

[70] V. Bamicha , P. Pergantis , C. Skianis , and A. Drigas , “Computational Neuroscience's Influence on Autism Neuro‐Transmission Research: Mapping Serotonin, Dopamine, Gaba, and Glutamate,” Biomedicines 13, no. 6 (2025): 1420. 40564138 10.3390/biomedicines13061420 PMC12189925

